# Increased Risk of Hypoglycemia Following Roux-en-Y Gastric Bypass Surgery in Patients Without Diabetes: a Propensity Score-Matched Analysis

**DOI:** 10.1007/s11695-024-07565-y

**Published:** 2024-11-13

**Authors:** Eman A. Toraih, Mohamed Doma, Aria Kaur Atwal, Benito Vlassis, Ahmed Abdelmaksoud, Hani Aiash, Runa Acharya

**Affiliations:** 1 Tulane University, New Orleans, LA USA; 2 Suez Canal University, Ismailia, Egypt; 3 Alexandria University, Alexandria, Egypt; 4 Columbia University, New York, NY USA; 5 Colgate University, Hamilton, NY USA; 6 University of California, Riverside, Riverside, CA USA; 7 SUNY Upstate Medical University, Syracuse, NY USA

**Keywords:** Roux-en-Y gastric bypass, Obesity, Hypoglycemia, Propensity score matching, Bariatric surgery

## Abstract

**Background:**

Roux-en-Y gastric bypass (RYGB) surgery is an effective treatment for obesity. However, the incidence and long-term risk of hypoglycemia after surgery in patients without diabetes remains unclear. This study aimed to investigate the prevalence of hypoglycemia following RYGB surgery in patients with obesity and without diabetes.

**Methods:**

A retrospective cohort study was conducted using the TriNetX database. The study population included 15,085 patients with obesity (BMI ≥ 30 kg/m^2^) who underwent RYGB surgery and 3,200,074 non-surgical controls, all without a history of diabetes or GLP-1 receptor agonist use. Propensity score matching was performed to balance baseline characteristics. The primary outcome was the incidence of hypoglycemia, defined by ICD-10-CM codes or laboratory values (glucose ≤ 70 mg/dL). Cox regression analysis was employed to calculate hazard ratios (HR) and 95% confidence intervals (CI).

**Results:**

In the overall study population, the risk of hypoglycemia was significantly higher in the RYGB group (18.70%, *n* = 2,810) compared to the control group (3.80%, *n* = 120,923; HR 4.3, 95% CI 4.14–4.46, *p* < 0.001). After propensity score matching (*n* = 14,916 per group), RYGB patients maintained an elevated risk (18.70%, *n* = 2,795) compared to matched controls (5.0%, *n* = 749; HR 3.7, 95% CI 3.44–4.05, *p* < 0.001). Time-series analysis revealed consistently higher hypoglycemia risk in the RYGB group, with hazard ratios ranging from 5.37 (95% CI 4.09–7.03) at 1 week to 3.75 (95% CI 3.45–4.06) at 10 years post-surgery (all *p* < 0.001). Subgroup analysis of RYGB patients who developed hypoglycemia showed a 30-day hospitalization rate of 21.3% and a mortality rate of 0.71%.

**Conclusion:**

RYGB surgery is associated with a significantly increased risk of hypoglycemia in patients with obesity and without diabetes, both in the short-term and long-term follow-up. These findings underscore the importance of monitoring and managing hypoglycemia in patients undergoing RYGB surgery, even in the absence of preexisting diabetes.

**Graphical Abstract:**

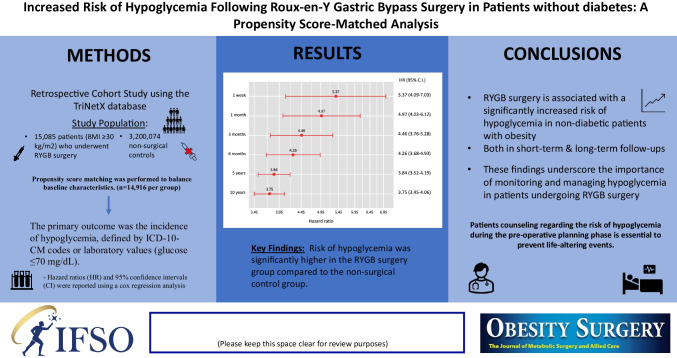

**Supplementary Information:**

The online version contains supplementary material available at 10.1007/s11695-024-07565-y.

## Introduction

Obesity is a worldwide health issue linked to several additional health problems, such as type 2 diabetes, cardiovascular disease, and specific types of cancer [1–3]. It is a global epidemic resulting from genetic susceptibility, increased availability of high-energy foods, and decreased physical activity, threatening global well-being [4]. Medical care, lifestyle modifications, food, and exercise are all available forms of treatment for obesity as well as Bariatric surgery which is considered an increasingly popular and highly successful option [5].

Roux-en-Y gastric bypass (RYGB) was first reported by Alan Wittgrove and Wesley Clark in 1994 [6], It combines restrictive and malabsorptive elements and is one of the most frequently performed surgical procedures for obesity in the USA [7, 8]. RYGB surgery has proven effective in treating severe obesity by causing substantial weight loss and enhancing related health issues [9, 10]. Nevertheless, the effects of RYGB surgery on the incidence of hypoglycemia, especially in individuals without diabetes, are still uncertain.

Hypoglycemia is a potentially serious complication that can occur after RYGB surgery, even in patients without a history of diabetes [11]. The reasons behind this may include changes in glucose metabolism, improved insulin sensitivity, and increased secretion of glucagon-like peptide-1 (GLP-1) after surgery [12, 13]. Despite the acknowledgment of this complication, the long-term risk of hypoglycemia following RYGB surgery in patients with obesity and without diabetes has not been thoroughly researched.

The aim of this study was to investigate the risk of hypoglycemia following RYGB surgery in patients with obesity and without a history of diabetes or GLP-1 receptor agonist use.

## Methods

### Data Source

This retrospective cohort study utilized the TriNetX database (TriNetX LLC, Cambridge, MA, USA), a global federated health research network providing access to electronic health records (EHRs). The TriNetX Global Collaborative network encompasses more than 275 million individuals across over 120 health care organizations (HCOs) worldwide, with established data usage and publication agreements. It offers comprehensive datasets including patient demographics, diagnoses, procedures, medications, and laboratory results, primarily collected from HCOs' EHRs. This extensive data collection ensures a holistic view of patient health and related outcomes, contributing to the robustness of our analyses. However, as with any EHR-based dataset, it may face typical data quality challenges such as incomplete or inaccurate entries, under-reporting of certain conditions, limited granularity, and potential data exclusions. To mitigate these issues, TriNetX employs rigorous data validation processes, including regular quality checks to identify and correct discrepancies, validation against external benchmarks for consistency and accuracy, and collaboration with data contributors to resolve issues and continuously improve data quality. The database provides continuous, comprehensive, up-to-the-month data, ensuring the most current information is available for analysis. For this study, the database was last accessed on September 22, 2024.

### Study Population

The study included patients aged 18 years or older with a body mass index (BMI) of 30 kg/m^2^ or higher. Patients who underwent RYGB surgery were identified using Current Procedural Terminology (CPT) codes 43,644 and 43,846. The non-surgical control group included patients with obesity who did not undergo bariatric surgery. Patients with a history of diabetes (ICD-10-CM codes E08-E13), GLP-1 receptor agonist use prior to the index date, or chromosomal and genetic disorders were excluded, Supplementary Table S1. The index date was defined as the date of RYGB surgery for the surgical group and a randomly assigned date for the non-surgical group.

### Study Variables

Baseline characteristics, including age, sex, race, and ethnicity, were extracted from the EHRs. Associated medical problems and medication use were assessed based on ICD-10-CM diagnosis codes and RxNorm medication codes, respectively.

### Procedural Characteristics

The study included patients who underwent two specific types of Roux-en-Y gastric bypass (RYGB) procedures:

-
  1. Laparoscopic gastric restrictive surgery combined with gastric bypass and a Roux-en-Y gastroenterostomy, where the Roux limb measures 150 cm or less.
  2. Gastric restrictive surgery with gastric bypass specifically for morbid obesity, also utilizing a short Roux-en-Y gastroenterostomy, defined as 150 cm or less.

This approach allows for a comprehensive understanding of the impact of surgical variations on patient results.

### Propensity Score Matching

To adjust for potential confounding factors, propensity score matching was performed using a 1:1 nearest-neighbor matching algorithm without replacement. Propensity scores were calculated using logistic regression, with treatment assignment (RYGB surgery vs. non-surgical control) as the dependent variable. Demographic factors included age, sex, race, and ethnicity. Socioeconomic determinants were represented by problems related to medical facilities and other health care, employment and unemployment, and housing and economic circumstances. Lifestyle factors comprised lack of physical exercise, problems related to sleep, overweight and obesity, dietary counseling, and smoking status. A range of associated medical problems were considered, including alcohol-related disorders, hypertensive diseases, metabolic disorders, malnutrition, vitamin deficiencies, acute and chronic kidney disease, hepatic failure, and neoplasms. Specific risk factors for hypoglycemia were also included: fibrosis and cirrhosis of liver, alcoholic liver disease, primary adrenocortical insufficiency, Addisonian crisis, hypopituitarism, severe sepsis with septic shock, and disorders of pyruvate metabolism and gluconeogenesis. Finally, the matching process accounted for medication use, specifically quinolones, glucagon, ACE inhibitors, and beta blockers. The matched cohorts were used for subsequent analyses.

### Outcomes of Interest

The primary outcome was the incidence of hypoglycemia, defined by the presence of ICD-10-CM diagnosis codes (E16.1, E16.2) or laboratory values (glucose ≤ 70 mg/dL) during the follow-up period. The time-to-event analysis was conducted for overall incidence and at various time points to assess both short-term and long-term risk. Short-term risk was evaluated at 1 week, 1 month, 3 months, and 6 months after the index date, while long-term risk was assessed at 5 years and 10 years after the index date.

### Subgroup Analysis

A subgroup analysis was conducted on 2,810 individuals from the surgery cohort who developed hypoglycemia. This analysis evaluated the impact on hospitalization rates and mortality within 30 days following a hypoglycemic event, as well as the length of stay for the first hospital admission. The aim was to quantify the acute clinical outcomes associated with hypoglycemic events in post-RYGB patients.

### Statistical Analysis

Baseline characteristics were compared between the RYGB surgery and non-surgical control groups using chi-square tests for categorical variables and t-tests for continuous variables. The prevalence of hypoglycemia was calculated in each group. Cox regression analysis was employed, and hazards ratio (HR) and 95% confidence intervals (CI) were reported. For the subgroup analysis, descriptive statistics were used to present hospitalization rates, length of hospital stay, and mortality in the surgery group. A two-sided* p*-value < 0.05 was considered statistically significant. All analyses were performed using the TriNetX platform.

## Results

### Characteristics of the Study Population

The study cohort comprised 15,085 patients who underwent RYGB surgery and 3,200,074 non-surgical controls (Table 1). Prior to propensity score matching, the RYGB surgery group was significantly younger (42.4 vs. 47.9 years, *p* < 0.001), had a higher proportion of females (79.2% vs. 54.6%, *p* < 0.001), and a larger percentage of white patients (73.1% vs. 62.5%, *p* < 0.001) compared to the non-surgical control group. Following propensity score matching, baseline characteristics were well-balanced between the RYGB surgery and non-surgical control groups (*n* = 14,916 per group; Table 1).

**Table 1:** Baseline characteristics of the study population before and after propensity score matching

Characteristics	Surgery (*n* = 15,085)	Non-surgery (*n* = 3,200,074)	*p*-value	Surgery (*n* = 14,916)	Non-surgery (*n* = 14,916)	*p*-value
Demographics						
Age at Index	42.4 ± 11.3	47.9 ± 17.1	< 0.001	42.5 ± 11.3	42.2 ± 13.3	0.33
Female	11,930 (79.2%)	1,736,423 (54.6%)	< 0.001	11,793 (79.1%)	11,946 (80.1%)	0.28
Male	2038 (13.5%)	1,095,300 (34.4%)		2038 (13.7%)	1913 (12.8%)	
White	11,011 (73.1%)	1,986,875 (62.5%)	< 0.001	10,877 (72.9%)	10,833 (72.6%)	0.56
Black or African American	1738 (11.5%)	434,843 (13.7%)		1737 (11.6%)	1795 (12%)	
Asian	52 (0.3%)	53,698 (1.7%)		52 (0.3%)	49 (0.3%)	
American Indian/Alaska Native	35 (0.2%)	12,734 (0.4%)		35 (0.2%)	45 (0.3%)	
Native Hawaiian/Pacific Islander	29 (0.2%)	38,159 (1.2%)		29 (0.2%)	102 (0.7%)	
Other race	393 (2.6%)	118,626 (3.7%)		390 (2.6%)	345 (2.3%)	
Unknown race	1810 (12%)	536,027 (16.9%)		1796 (12%)	1747 (11.7%)	
Not Hispanic or Latino	11,102 (73.7%)	2,040,241 (64.1%)	< 0.001	11,020 (73.9%)	9815 (65.8%)	0.38
Hispanic or Latino	1804 (12%)	338,014 (10.6%)		1754 (11.8%)	2763 (18.5%)	
Socioeconomic determinants						
Problems related to medical facilities and other health care	10 (0.1%)	2785 (0.1%)	0.38	10 (0.1%)	11 (0.1%)	0.82
Problems related to employment and unemployment	65 (0.4%)	9533 (0.3%)	0.003	64 (0.4%)	81 (0.5%)	0.15
Problems related to housing and economic circumstances	140 (0.9%)	18,349 (0.6%)	< 0.001	139 (0.9%)	154 (1%)	0.37
Lifestyle factors						
Lack of physical exercise	46 (0.3%)	1314 (0.0%)	< 0.001	45 (0.3%)	48 (0.3%)	0.75
Problems related to sleep	117 (0.8%)	6728 (0.2%)	< 0.001	117 (0.8%)	98 (0.7%)	0.19
Dietary counseling	5927 (39.3%)	42,780 (1.3%)	< 0.001	5775 (38.7%)	5734 (38.4%)	0.62
Smoking	606 (4%)	89,848 (2.8%)	< 0.001	602 (4%)	583 (3.9%)	0.57
Associated medical problems						
Alcohol related disorders	277 (1.8%)	71,052 (2.2%)	0.001	276 (1.9%)	219 (1.5%)	0.11
Hypertensive diseases	7142 (47.4%)	822,051 (25.8%)	< 0.001	7010 (47%)	7031 (47.1%)	0.80
Metabolic disorders	6264 (41.6%)	785,294 (24.7%)	< 0.001	6202 (41.6%)	6157 (41.3%)	0.59
Malnutrition	212 (1.4%)	13,264 (0.4%)	< 0.001	187 (1.3%)	198 (1.3%)	0.57
Vitamin deficiencies	5509 (36.6%)	290,107 (9.1%)	< 0.001	5368 (36%)	5248 (35.2%)	0.14
Acute & chronic kidney disease	355 (2.4%)	114,291 (3.6%)	< 0.001	355 (2.4%)	288 (1.9%)	0.08
Hepatic failure	15 (0.1%)	7353 (0.2%)	0.001	15 (0.1%)	13 (0.1%)	0.70
Neoplasms	3205 (21.3%)	567,055 (17.8%)	< 0.001	3198 (21.4%)	3129 (21%)	0.33
Risk factors for hypoglycemia						
Fibrosis and cirrhosis of liver	98 (0.7%)	20,971 (0.7%)	0.89	97 (0.7%)	83 (0.6%)	0.29
Alcoholic liver disease	11 (0.1%)	9957 (0.3%)	< 0.001	11 (0.1%)	22 (0.1%)	0.06
Primary adrenocortical insufficiency	15 (0.1%)	2186 (0.1%)	0.15	15 (0.1%)	18 (0.1%)	0.60
Addisonian crisis	13 (0.1%)	1754 (0.1%)	0.10	13 (0.1%)	14 (0.1%)	0.84
Hypopituitarism	17 (0.1%)	3557 (0.1%)	0.97	17 (0.1%)	11 (0.1%)	0.25
Severe sepsis with septic shock	14 (0.1%)	4721 (0.1%)	0.07	14 (0.1%)	22 (0.1%)	0.18
Disorders of pyruvate metabolism and gluconeogenesis	10 (0.1%)	270 (0%)	< 0.001	10 (0.1%)	10 (0.1%)	1.0
Medications						
Quinolones	2122 (14.1%)	318,633 (10%)	< 0.001	2117 (14.2%)	2123 (14.2%)	0.92
Glucagon	197 (1.3%)	39,902 (1.3%)	0.56	197 (1.3%)	166 (1.1%)	0.10
ACE inhibitors	1592 (10.6%)	286,625 (9%)	< 0.001	1589 (10.7%)	1546 (10.4%)	0.41
Beta blockers	3065 (20.3%)	438,226 (13.8%)	< 0.001	3030 (20.3%)	2829 (19%)	0.30

Data are presented as mean ± standard deviation or frequency (percentage). *P*-values were calculated using t-tests for continuous variables and chi-square tests for categorical variables. Bold values indicate statistical significance (*p* < 0.05)

### Risk of Hypoglycemia in the Overall Study Population

**Fig. 1:**
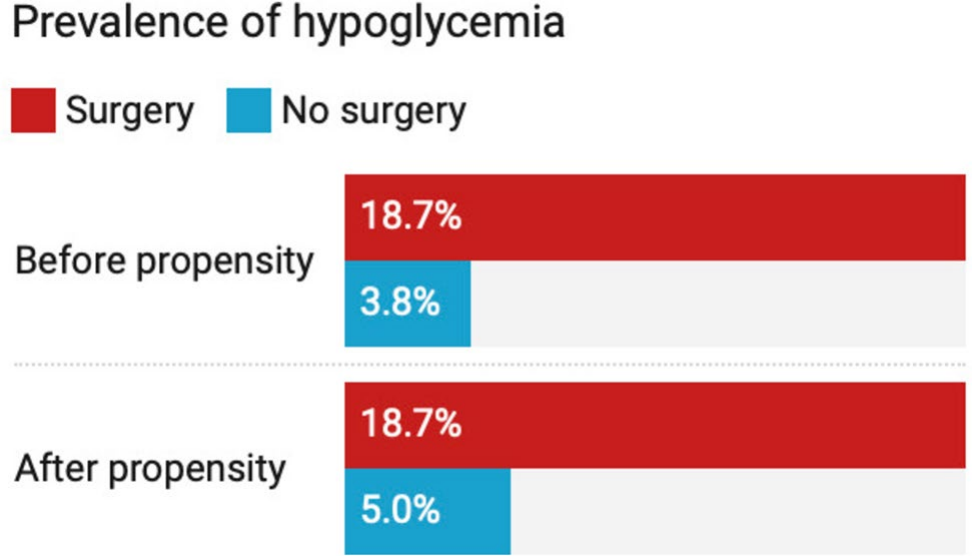
Prevalence of hypoglycemia in the overall study population before and after propensity score matching. The risk of hypoglycemia was significantly higher in the RYGB surgery group compared to the non-surgical control group, both before and after propensity score matching (*p* < 0.001)

In the overall population, the risk of hypoglycemia was substantially higher in the RYGB group (18.70%, *n* = 2,810) compared to control group (3.80%, *n* = 120,923), with patients who underwent RYGB surgery demonstrated a four-fold increased risk of hypoglycemia (HR 4.3, 95% CI 4.14–4.46, *p* < 0.001). This trend persisted after propensity score matching, with the RYGB group maintaining an elevated risk (18.70%, *n* = 2,795) compared to matched controls (5.0%, *n* = 749). Post-matching analysis revealed that RYGB surgery patients had nearly a three-fold increased risk of hypoglycemia compared to matched controls (HR 3.7, 95% CI 3.44–4.05, *p* < 0.001) (Fig. 1).

### Time-Series Analysis

**Fig. 2:**
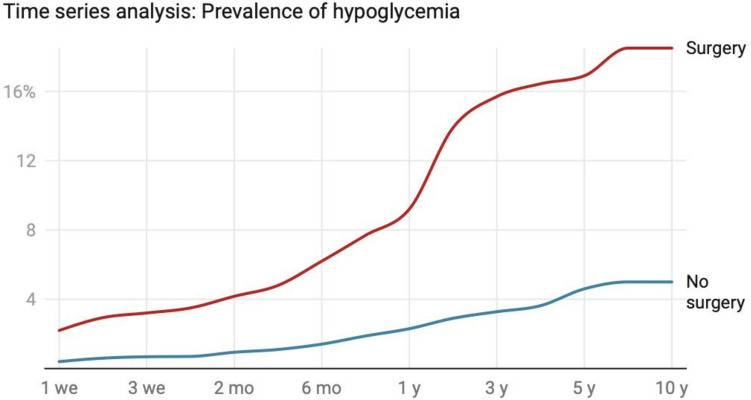
Time-series follow-up of propensity score-matched cohorts. **A** Incidence of hypoglycemia at various time points following RYGB surgery compared to non-surgical controls. The incidence of hypoglycemia remained significantly elevated in the RYGB surgery group throughout the 10-year follow-up period (all *p* < 0.001)

**Fig. 3:**
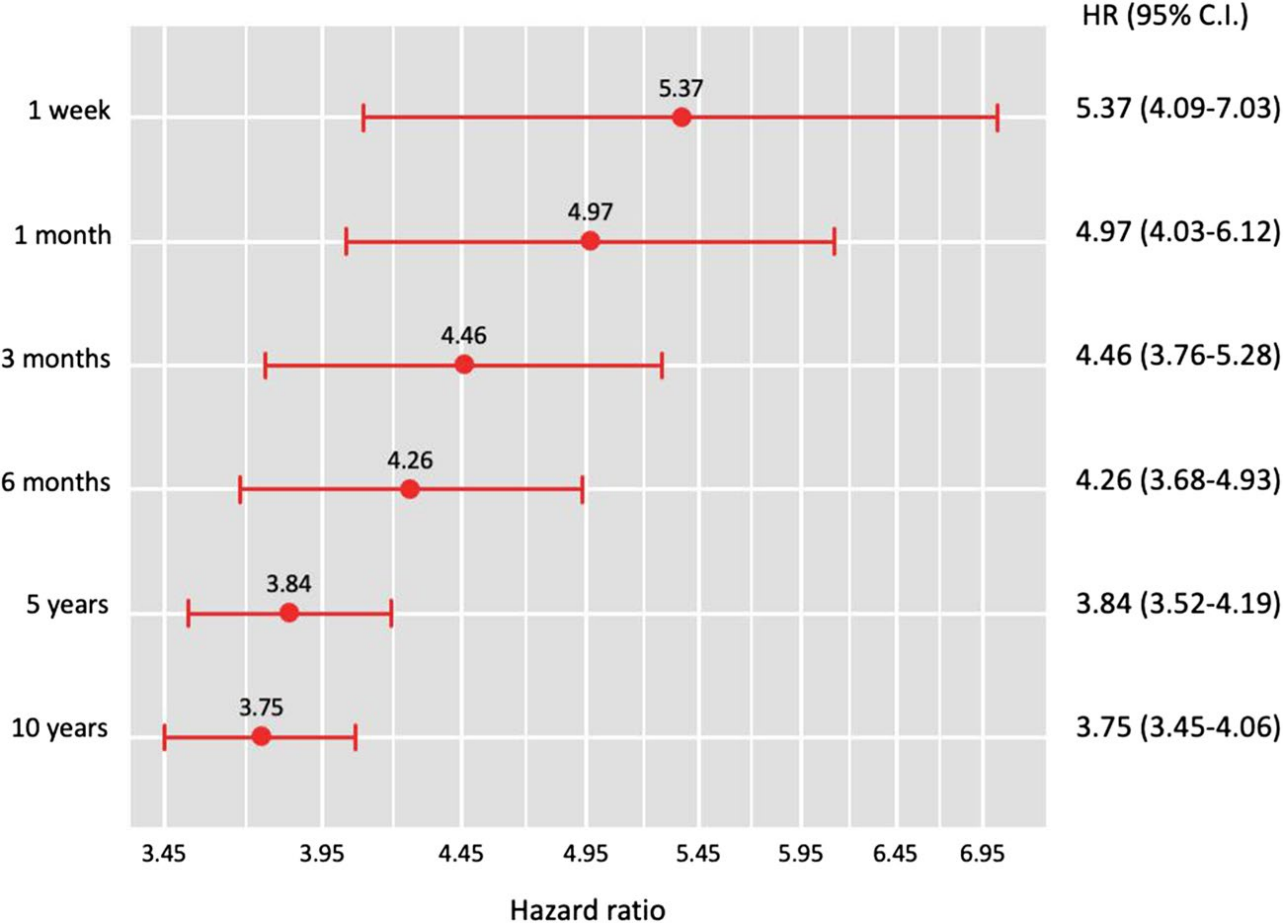
Risk of hypoglycemia at each time point following RYGB or obesity diagnosis. Cox regression analysis was employed, and hazards ratio (HR) and 95% confidence intervals (CI) are reported. The risk of hypoglycemia remained significantly elevated in the RYGB surgery group throughout the 10-year follow-up period (all *p* < 0.001)

Time-series analysis of matched cohorts revealed a consistently higher hypoglycemia risk in the RYGB group across all time points (Fig. 2). Cox regression analysis demonstrated elevated hazard ratios from 1 week (HR 5.37, 95% CI 4.09–7.03) to 10 years (HR 3.75, 95% CI 3.45–4.06) post-surgery (all *p* < 0.001; Fig. 3). The RYGB group exhibited a rapid increase in hypoglycemia incidence during the first 6 months (2.20% to 6.20%), followed by a slower but steady rise to 10 years (18.50%), while the non-surgical group showed a much slower increase (0.40% to 5.00% over 10 years). Although the hazard ratio was highest immediately post-operation, its persistent elevation after a decade underscores the long-term impact of RYGB surgery on hypoglycemia risk.

### Subgroup Analysis

A subgroup analysis was conducted on the 2,810 individuals from the RYGB cohort who developed hypoglycemia to quantify the acute clinical outcomes associated with hypoglycemic events. Within 30 days of a hypoglycemic event, 21.3% (*n* = 598) of these patients were hospitalized. The median length of stay for the first hospital admission was 1.5 days (IQR: 1–3 days). The 30-day mortality rate following a hypoglycemic event was 0.71% (*n* = 20).

## Discussion

In this large-scale, propensity score-matched analysis, we found that RYGB surgery is associated with a significantly increased risk of hypoglycemia in patients with obesity and without diabetes. The risk was observed as early as 1-week post-surgery and persisted throughout the 10-year follow-up period, with hazard ratios ranging from 5.37 at 1 week to 3.75 at 10 years post-surgery.

These findings build on the pre-existing knowledge that RYGB does in fact increase the risk of post-surgical hypoglycemia among patients with obesity significantly. Our results show an approximately 3.7-fold increase in risk among the propensity-score matched cohort, which although lower than some previous estimates, is still consistent with other studies showing increased risk of hypoglycemia postoperative [14].

The increased risk of hypoglycemia following RYGB may be attributed to several factors, including increased glycemic variability, altered glucose metabolism, increased insulin sensitivity, and enhanced glucagon-like peptide-1 (GLP-1) response which are particularly evident at 1 and 2 year time points [15, 16]. Another potential cause may be delayed improvement in pancreatic β-cell sensitivity to changes in circulating glucose, which was evidently observed in patients who had a higher decrease in BMI during the first 12 months post-surgery [17].

Among all potential complications of RYGB, incidence of hypoglycemia, considered low in some studies [18, 19], might be slightly misleading. This is due to the desired effects of hypoglycemia among patients with diabetes who undergo RYGB. However, our results show an 18.70% prevalence of hypoglycemia in the RYGB group compared to 5.0% in the matched control group. Our subgroup analysis further revealed that among RYGB patients who experienced hypoglycemia, 21.3% were hospitalized within 30 days of the event, with a median hospital stay of 1.5 days. Moreover, the 30-day mortality rate following a hypoglycemic event was 0.71%, underscoring the potential severity of this complication. Despite the lack of literature supporting this, Vaurs et. al [20] have recognized the risk of hypoglycemia post-surgery and the longer duration patients spend under 3.3 mmol/L. This was attributed to a certain patients’ phenotype which generally leads to increased susceptibility to hypoglycemia. These patients should be carefully monitored post-RYGB.

Appropriate management of hypoglycemia post-RYGB should be given further consideration in the future to prevent multiple secondary complications. This can include changes in brain responses to hypoglycemia that may exacerbate hypoglycemia due to reduced insulin-antagonistic reactions. Neural activation in critical brain regions, including the hypothalamus, plays a key role in coordinating glucose regulation [21]. Furthermore, hypoglycemia is associated with dementia, macrovascular complications, falls and fractures, and cardiovascular death particularly in older patients and most importantly may ultimately increase mortality [22].

Well-illustrated treatment strategies targeting appropriate management for post-RYGB hypoglycemia in patients without diabetes are still missing in the literature. However, several proposed dietary, pharmacologic and even surgical approaches can be considered [23]. Surgical interventions such as reversal of RYGB and pancreatectomy have reported success rates, with trends leaning towards higher effectiveness with reversal surgery [24]. A clear outline for the most optimal treatment strategy is yet to be defined.

### Future Directions

These findings highlight the importance of long-term monitoring and management of hypoglycemia in patients undergoing RYGB surgery, especially in the absence of preexisting diabetes. The consistently elevated risk of hypoglycemia throughout the 10-year follow-up period emphasizes the need for ongoing vigilance. Pre-operative assessment for patients' liability to hypoglycemia should also be essential along with proper patient education about the possibility of such a complication. Furthermore, the significant rate of hospitalization and mortality associated with hypoglycemic events in our subgroup analysis underscores the need for prompt recognition and management of this complication.

### Strengths and Limitations

The study's strengths include the large sample size, long-term follow-up, and use of propensity score matching to control for potential confounding factors. Additionally, our subgroup analysis provides valuable insights into the acute clinical outcomes associated with hypoglycemic events in post-RYGB patients. However, the study also has some limitations. First, the retrospective nature of the study may introduce bias due to unmeasured confounding factors. Second, the definition of hypoglycemia based on ICD-10-CM codes and laboratory values may not capture all cases, potentially underestimating the true incidence of hypoglycemia. Moreover, due to the nature of the TriNetX database, which provides aggregated data rather than individual patient information, our ability to investigate specific reasons for each hospitalization along with directly assessing missing or incomplete data at the individual patient level was limited. Finally, while our subgroup analysis provides some insight into the clinical impact of hypoglycemic events, we were unable to assess the full spectrum of severity and long-term consequences of these events.

## Conclusion

In conclusion, while RYGB surgery remains an effective treatment for severe obesity, the increased risk of hypoglycemia should be carefully considered in the decision-making process and post-operative care of patients with obesity and without diabetes. Balancing the benefits of weight loss and metabolic improvements with the potential risk of hypoglycemia is crucial for optimizing patient outcomes and ensuring the long-term success of bariatric surgery.

## Supplementary Information

Below is the link to the electronic supplementary material.

Supplementary file1 (DOCX 16 KB)

## Data Availability

No datasets were generated or analysed during the current study.
