# Supplementary material for: Increased Risk of Hypoglycemia Following Roux-en-Y Gastric Bypass Surgery in Patients Without Diabetes: a Propensity Score-Matched Analysis
Source: Obes Surg. 2024 Nov 13;34(12):4385–92. doi: 10.1007/s11695-024-07565-y (PMC11671553; doi:10.1007/s11695-024-07565-y)
Supplement: Supplementary file 1 — Supplementary file1 (DOCX 16 KB) [file 11695_2024_7565_MOESM1_ESM.docx]

**Supplementary materials**

**Supplementary Table S1**. Diagnosis and procedure codes used for patient recruitment

| **Category** | **Code** | **Description** |
| --- | --- | --- |
| **Inclusion criteria** |  |  |
| ***Obesity*** |  |  |
| Diagnosis | UMLS:ICD10CM:Z68.3 | Body mass index [BMI] 30-39, adult |
| Diagnosis | UMLS:ICD10CM:Z68.4 | Body mass index [BMI] 40 or greater, adult |
| ***Bariatric surgery*** |  |  |
| Procedure | UMLS:CPT:43644 | Laparoscopy, surgical, gastric restrictive procedure; with gastric bypass and Roux-en-Y gastroenterostomy (roux limb 150 cm or less) |
| Procedure | UMLS:CPT:43846 | Gastric restrictive procedure, with gastric bypass for morbid obesity; with short limb (150 cm or less) Roux-en-Y gastroenterostomy |
| **Exclusion criteria** |  |  |
| ***Diabetes*** |  |  |
| Diagnosis | UMLS:ICD10CM:E08-E13 | Diabetes mellitus |
| ***GLP1-R agonists*** |  |  |
| Medication | NLM:RXNORM:2601723 | Tirzepatide |
| Medication | NLM:RXNORM:1991302 | Semaglutide |
| Medication | NLM:RXNORM:1551291 | Dulaglutide |
| Medication | NLM:RXNORM:475968 | Liraglutide |
| Medication | NLM:RXNORM:1440051 | Lixisenatide |
| Medication | NLM:RXNORM:1534763 | Albiglutide |
| Medication | NLM:RXNORM:60548 | Exenatide |
| **Outcomes** |  |  |
| ***Hypoglycemia*** |  |  |
| Diagnosis | UMLS:ICD10CM:E16.2 | Hypoglycemia, unspecified |
| Diagnosis | UMLS:ICD10CM:E16.1 | Other hypoglycemia |
| Laboratory | UMLS:LNC:2345-7 | Glucose [Mass/volume] in Serum or Plasma (at most 70.00 mg/dL (most recent occurrence)) |
| Laboratory | TNX:9025 | Glucose [Mass/volume] in Serum, Plasma or Blood (at most 70.00 mg/dL (most recent occurrence)) |
| Laboratory | UMLS:LNC:2339-0 | Glucose [Mass/volume] in Blood (at most 70.00 mg/dL (most recent occurrence)) |
